# A Computational Model for Understanding Stem Cell, Trophectoderm and Endoderm Lineage Determination

**DOI:** 10.1371/journal.pone.0003478

**Published:** 2008-10-22

**Authors:** Vijay Chickarmane, Carsten Peterson

**Affiliations:** 1 Division of Biology, California Institute of Technology, Pasadena, California, United States of America; 2 Computational Biology and Biological Physics, Department of Theoretical Physics, Lund University, Lund, Sweden; 3 Lund Strategic Research Center for Stem Cell Biology and Cell Therapy, Lund University, Lund, Sweden; University of Glasgow, United Kingdom

## Abstract

**Background:**

Recent studies have associated the transcription factors, Oct4, Sox2 and Nanog as parts of a self-regulating network which is responsible for maintaining embryonic stem cell properties: self renewal and pluripotency. In addition, mutual antagonism between two of these and other master regulators have been shown to regulate lineage determination. In particular, an excess of Cdx2 over Oct4 determines the *trophectoderm* lineage whereas an excess of Gata-6 over Nanog determines differentiation into the *endoderm* lineage. Also, under/over-expression studies of the master regulator Oct4 have revealed that some self-renewal/pluripotency as well as differentiation genes are expressed in a biphasic manner with respect to the concentration of Oct4.

**Methodology/Principal Findings:**

We construct a dynamical model of a minimalistic network, extracted from ChIP-on-chip and microarray data as well as literature studies. The model is based upon differential equations and makes two plausible assumptions; activation of Gata-6 by Oct4 and repression of Nanog by an Oct4–Gata-6 heterodimer. With these assumptions, the results of simulations successfully describe the biphasic behavior as well as lineage commitment. The model also predicts that reprogramming the network from a differentiated state, in particular the *endoderm* state, into a stem cell state, is best achieved by over-expressing Nanog, rather than by suppression of differentiation genes such as Gata-6.

**Conclusions:**

The computational model provides a mechanistic understanding of how different lineages arise from the dynamics of the underlying regulatory network. It provides a framework to explore strategies of reprogramming a cell from a differentiated state to a stem cell state through directed perturbations. Such an approach is highly relevant to regenerative medicine since it allows for a rapid search over the host of possibilities for reprogramming to a stem cell state.

## Introduction

Recent breakthroughs in reprogramming differentiated cells into embryonic stem cells [1], [2], [3], [4], [5], have made major inroads into stem cell biology. What emerges is a relatively small core of master regulators that are required for successful reprogramming of a differentiated cell into a cell exhibiting stem cell like properties. This set of transcription factors (TF) has previously been established as candidates to regulate both pluripotency and differentiation of embryonic stem cells [6], [7], [8], [9], [10].

The fact that there appears to be only a hand full of master regulators argues for a computational approach. A model based upon regulatory mechanisms inferred from ChIP-on-chip and microarray data can quantify functionality of the genetic network. This would also provide a platform for reprogramming studies, by allowing us to enumerate the possibilities of over/under-expression of key TFs. The motivation for this model comes from a recent review [9], in which lineage determination, *i.e.* how pluripotency and self-renewal versus the two differentiation lineages, *trophectoderm* and *endoderm*, arise as a result of the system finding different stable states. These are given by combinations of certain TF concentrations, resulting from the dynamics of the interaction network, which contains several positive and negative feedback loops. At the core of the network reside Oct4, Sox2 and Nanog, which form a self-organized core of the TFs maintaining pluripotency and self-renewal [6], [7], [8]. A computational model of the dynamics of this core network has revealed that it functions as a bistable switch, which in the on state, corresponds to all these TFs being expressed and the downstream differentiation target genes being shut off [11].

In this work we develop a dynamical model of lineage determination based upon a minimal circuit, as discussed in [9], which contains the Oct4/Sox2/Nanog core as well its interaction with a few other key genes. The model dynamics both suggests the mechanisms of interaction as gleaned from data, as well as point to reprogramming strategies.

The *trophectoderm* lineage arises from the balance between Oct4 and Cdx2 through mutual antagonism; an excess of Cdx2 gives rise to the *trophectoderm* lineage, whereas an excess of Oct4 results in the stem cell state [12]. The *endoderm* lineage is also conjectured to result from mutual antagonism between Nanog and Gata-6; an excess of Gata-6 pushes the cell into the *endoderm* lineage [13]. Microarray studies of cells in which Oct4 is over/under-expressed [14], reveal an interesting result. A set of genes, which determine differentiation, are expressed at low and high levels of Oct4, whereas never in the intermediate range. On the other hand several genes responsible for the stem cell state, are expressed only for an intermediate concentration of Oct4. It is a challenge to understand the origin of such a “bell/inverse bell shaped” [14] expression behaviour of these TFs as functions of Oct4. Similar observations were indicated in earlier studies [15], [16]. The picture that emerges is that pluripotency is the default state when Oct4 and the other two core components, Sox2 and Nanog, are “held” together at some intermediate range of concentration. Over-expression of Oct4, pushes the system into the *endoderm* lineage. In contrast, the *trophectoderm* lineage arises when Oct4 is suppressed and Cdx2 develops a high level. Hence, the relative levels of the core TFs determine three stable states [16]. To reprogram the cell from one state to the other, the stable states have to be toggled, by applying a particular perturbation (expressing a particular gene). First we review the core embryonic stem cell network, consisting of Oct4, Sox2 and Nanog. We then expand this core by including interactions of these genes with Cdx2, Gata-6 and Gcnf. The specific additional assumptions required in order to obtain the “bell/inverse bell shaped” curve for the expression of the network components are then discussed. These assumptions are then incorporated into a computational model for the extended embryonic stem cell network. Finally, we probe this system with regard to different perturbations which address reprogramming strategies.

## Results

### The embryonic stem cell circuit

**Figure 1:**
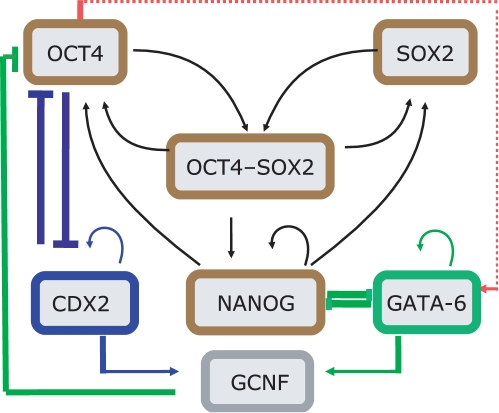
The key TF interactions in the embryonic stem cell circuit. The *trophectoderm* lineage is determined by the antagonism between Oct4 and Cdx2, whereas the balance between Gata-6 and Nanog determines the *endoderm* lineage. The dashed red line indicates an interaction which emerges out of ChIP-chip data. It is also supported by the phenomenological observation that over-expression of Oct4 ultimately leads to the *endoderm* lineage, in which Gata-6 is strongly expressed.

In [11] a dynamical model was developed for the core embryonic stem cell network which comprises Oct4, Sox2 and Nanog. It was found that cooperative interactions between these TFs give rise to a bistable switch-like behavior. One key prediction of the resulting dynamics is that over-expression of Nanog can maintain pluripotency of the cell even in the absence of the external factor(s) inducing Oct4 and Sox2. This result is consistent with experiments for mouse embryonic stem cells [8]. In [12], the authors discussed the mutual antagonism between Cdx2 and Oct4 which determines the *trophectoderm* versus stem cell fate. The heterodimer Cdx2-Oct4 binds to both Cdx2 and Oct4 acting as a repressor. Since Cdx2 and Oct4 are both autoregulatory, the latter through the Oct4/Sox2 complex, an excess of Cdx2 will give rise to the *trophectoderm* lineage, and similarly an excess of Oct4 defines the stem cell lineage. Therefore, with respect to an external signal which regulates the Oct4, low values of this signal would correspond to the *trophectoderm* state. On the other hand, the mutual antagonism between Gata-6 and Nanog decides between *endoderm* and stem cell fates [13]. An excess of Gata-6 leads to the *endoderm* fate. The master regulator Oct4 also receives negative feedback from Gcnf [17], [18], which itself is activated by both Gata-6 and Cdx2 [9]. This negative feedback ensures that once differentiated, the pluripotency genes are shut off. The assembled network interactions are displayed in Figure 1. The red dotted line, indicates that Oct4 positively induces Gata-6, and is a hypothesis, which arises due to a dynamical consideration of the model as will be discussed below. What is known from ChIP-on-chip experiments is that Gata-6 is a target of both Nanog and Oct4 [6], [19].

From Figure 1 it is not intuitively obvious that the decisions implemented by the two different mutual antagonistic interaction pairs Cdx2/Oct4 and Gata-6/Nanog give rise to the *trophectoderm* and *endoderm* lineages with the default state being the pluripotent embryonic one, where the latter is decided by the Oct4/Sox2/Nanog switch. Two questions come to mind: (i) What are the specific combinations of TFs that determine a particular lineage and (ii) how do the different genes toggle between high and low expression levels. Moreover, microarray results [14], show that certain genes are expressed in a “bell/inverted bell shaped” manner with respect to the Oct4 concentration. Hence the exact mechanisms of activation/repression must be able to explain this important finding.

### A network description of the “bell shaped” curve

In this section we focus on the “bell/inverted bell shape” (biphasic response) of GATA-6 [14] as a function of Oct4 concentration, and discuss what type of interactions between Oct4, Nanog and Gata-6, can give such dynamics? In the next section we construct the full network by including these inferred regulatory mechanisms. In [20], the authors discuss a squelching mechanism between Oct4 and a co-factor, which can give rise to a biphasic behavior of target gene which is jointly regulated by Oct4 and the co-factor. In Supplementary S1 we investigate a simple model realization of this mechanism, where we argue that the squelching mechanism by itself is not sufficient to provide the biphasic behavior (see Figure S3, Figure S4, Figure S5 and Table S1). Rather, we argue for a network-like mechanism by which biphasic behavior can be obtained [9].

**Figure 2:**
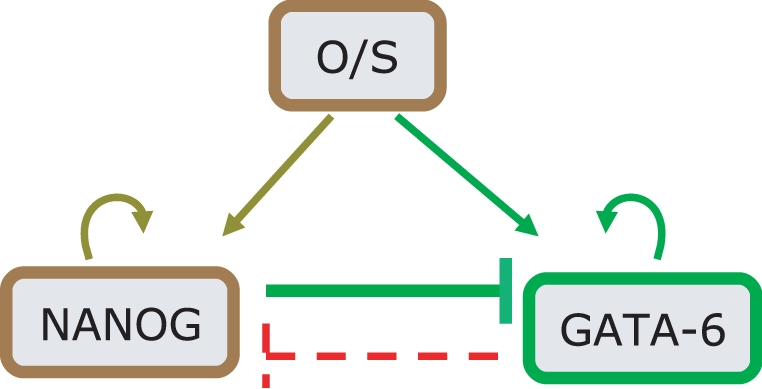
The essential TF interactions between Gata-6 and Nanog, which determine the *endoderm* lineage. In this condensed motif, the factor O/S represents both Oct4-Sox2 as well as Oct4. Both Nanog and Gata-6 are positively induced by Oct4. The dashed red line indicates a hypothesis, which emerges as a necessity from a model analysis (see Main Text).

**Figure 3:**
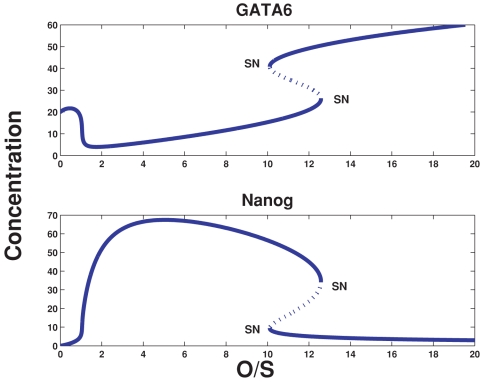
The steady state values of Gata-6 and Nanog as a function of the input O/S. Whereas Nanog displays the “bell shaped” curve, Gata-6 displays the “inverted bell shaped” curve. The steady state curves also show two saddle-node (SN) bifurcations, indicating a bistable state, or hysteresis. The bistability arises due to the cooperative effects between autoregulation of Gata-6 and the repression of Nanog by Gata-6-Oct4. The dotted line indicates the unstable states.

Extracting from Figure 1, the interactions between Oct4, Nanog and Gata-6, we deduce the simple motif displayed in Figure 2, which shows *O*/*S*, a proxy for Oct4 or Oct4-Sox2, that activates both Nanog and Gata-6 (the latter is assumed, since over-expression of Oct4 leads to induction of Gata-6 [14]). Also shown is the mutual antagonism between Gata-6 and Nanog, as well as the Gata-6 and Nanog positive self-interactions. From Figure 2, we argue that for low *O*/*S*, when Nanog is not fully turned on, the default state is that Gata-6 is on. This is to be expected since, Gata-6 is auto-regulating [9], and hence can maintain stable levels. Thereafter, increasing *O*/*S*, should lead to activation of Nanog, such that the latter increases its levels, and at some threshold of *O*/*S*, switches Gata-6 off. If we now demand, that GATA-6 exhibits biphasic response with respect to *O*/*S*, then as *O*/*S* continues to increase, since Gata-6 must be somehow switched on, Nanog must be switched off. It seems inconsistent however, that *O*/*S*, which induces Nanog, can switch Gata-6 on, where the latter itself is suppressed by Nanog. One mechanism, however, that could give rise to this, is if we assume that Nanog is suppressed by the heterodimer, *O*/*S*–Gata-6. This leads to the following consequence: At the higher threshold of *O*/*S*, when Gata-6 levels begin to increase, the heterodimer *O*/*S*–Gata-6 suppresses Nanog, thereby, allowing Gata-6 to ultimately switch on. Translating these assumptions into mathematical terms, we describe the evolution of Nanog and Gata-6 concentration levels as the ordinary differential equations (Eq. 1) given in *Materials and Methods*. In Figure 3, the steady state curves (which are obtained by setting the right hand side of Eq. 1 to zero) for Nanog and Gata-6 reflect the biphasic behavior with respect to the concentration of *O*/*S*. The steady state plot also shows a hysteretic behavior, which arises essentially due to the cooperative effect of autoregulation of Gata-6, and suppression of Nanog. Hence, this simple model can help explain the regulation required between the mutually antagonistic pair like Nanog/Gata-6, such that Nanog displays a “bell shaped” curve, whereas, Gata-6 displays the “inverted bell shaped” curve.

### The stem cell, trophectoderm and endoderm lineages

Assembling the entire network shown in Figure 1, we obtain equations for the TF concentrations given in *Materials and Methods* (Eq. 2). In particular we study the combinations of TFs expressed as functions of the Oct4 concentration, by assuming that an external factor *A* induces Oct4.

#### The trophectoderm state

**Figure 4:**
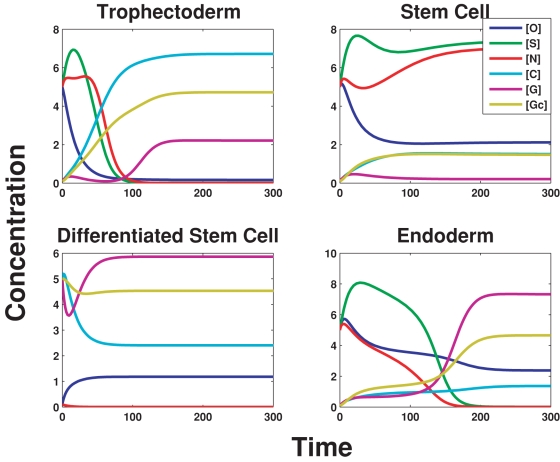
Time series concentrations of Oct4, Sox2, Nanog, Cdx2, Gata-6 and Gcnf for the three regimes (in terms of the concentration levels of A), indicating the final steady state values. The *trophectoderm* and *endoderm* lineages are the only possible states of the system for low and high *A*. However, for intermediate *A*, the initial conditions of Oct4/Sox2/Nanog determine the final steady state as can be seen for upper right and lower left, which give either the embryonic or differentiated stem cell (*endoderm*) lineage, depending on whether the initial conditions were relatively high/low values of [*O*],[*S*]&[*N*]. The system is bistable, and hence can choose either of the two states depending on the initial conditions (see Figure S1).

For low values of *A*, the external signal activating Oct4, the Oct4/Sox2/Nanog switch fails to turn on, and the balance between Oct4 and Cdx2 tips in favor of Cdx2. Moreover, since Cdx2 is autoregulating, the latter is able to maintain itself. Gcnf, which is activated by Cdx2 ensures that Oct4 is kept repressed. Another interesting feature in this region is that Gata-6 is expressed [21]. This occurs since Nanog, which represses Gata-6, is itself off. Furthermore, Gata-6 is autoregulatory and hence remains stable at high levels. In Fig. 4 (upper left), a time series is displayed for *A* = 1, which shows the steady states being achieved from initial conditions such that Oct4, Sox2 and Nanog are high and all other TF concentrations are low. With *A* being low, the stem cell is switched off, and hence Cdx2, Gcnf and Gata-6 reach relatively high levels.

#### The stem cell state

For *A* within an intermediate range, Oct4 is activated, and hence the stem cell “box” Oct4/Sox2/Nanog switches on as can be seen in Figure 4 (upper right) for *A* = 10 (Sox2/Nanog levels are relatively higher than Cdx2/Gata-6). Since Oct4 and Nanog suppress Cdx2 and Gata-6 respectively, there is no repressive feedback on Oct4 through Gcnf. This region is bistable, as can be seen in Figure 4 (lower left) for *A* = 10, which shows that if the initial conditions are chosen such that Oct4/Sox2/Nanog are initially at low levels, then the system does not reach the stem cell state, and infact Gcnf/Gata-6 and to some extent Cdx2 are at higher levels.

#### The endoderm state

For yet higher values of *A*, the Oct4 levels are sufficiently high to induce Gata-6, which ultimately shuts down Nanog as is clear from Figure 4 (lower right) for *A* = 25. This in turn weakens the positive feedback to Oct4 and Sox2, which is therefore unable to maintain Sox2. At the same time, Cdx2 is kept suppressed by the over-expression of Oct4. Hence, only Oct4, Gata-6 and Gcnf are on. Gcnf represses Oct4 to some extent. However, since Oct4 is activated by a large value of *A*, this effect is minimal. Finally, a consideration of the stable values of all the TFs, over the entire range of *A* (see Figure S1), shows that Gata-6 and Gcnf are expressed in an “inverted bell shaped” curve with respect to Oct4, whereas Sox2 and Nanog are expressed strongly in an intermediate range of *A* (“bell shaped” curve). The network dynamics of these master regulators therefore suggests three *stable* regimes, corresponding to the three lineages. The external factors determine which state the system will go into. The “bell/inverted bell shaped” curve displayed by the expression levels of several self-renewal/differentiation genes, found in [14] can now be hypothesized to arise out of these basic interactions. This is because many of the target genes are regulated singly as well as jointly by the master regulators Oct4, Sox2 and Nanog. Since the latter are themselves expressed in a “bell shaped” curve, it seems reasonable that they would regulate genes as “bell/inverted bell” if they are activators/repressors respectively.

### A strategy for reprogramming

**Figure 5:**
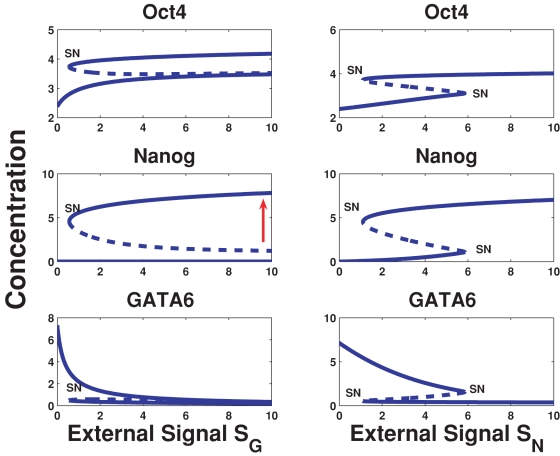
Left: Steady state concentrations of Oct4, Nanog and Gata-6 as functions of an external signal *S_G_*, which represses Gata-6. Although GATA-6 levels decrease, as *S_G_* increases, Nanog and Sox2 fail to get induced (unless an external perturbation on Nanog is applied: red arrow) and hence the default embryonic state is not achieved. Right: Steady state concentrations of Oct4, Nanog and Gata-6 as functions of the external signal *S_N_* which induces Nanog. Induction of Nanog leads to the reinforcement of the Oct4-Sox2-Nanog sub-system, due to their shared positive feedback regulations: Nanog therefore shuts down GATA-6, and ultimately the embryonic state is attained.

One application of dynamical modeling is to probe the effects from perturbations on the network. Given that the system is in a particular state, for example the *endoderm* state, one can ask which type of perturbation is required to reprogram it to the stem cell state. More precisely, in the *endoderm* state, Gata-6, Gcnf and Oct4 are expressed. To reach the stem cell state, two possible paths are: (i) Suppression of Gata-6, or (ii) activation of Nanog. To describe these two options quantitatively, we modify the expressions for d[N]/dt and d[G]/dt in Eq. 2, as described in *Materials and Methods*. The suppression of Gata-6 is modeled by including an external factor *S_G_*, which has the effect of repressing Gata-6. The panels of the left columns in Figure 5 shows the steady state values of Oct4, Nanog and Gata-6 as functions of the signal *S_G_*. Although Gata-6 is successfully repressed, and this in principle should allow Nanog to increase, Nanog continues to be at low levels, since there is not enough activation into Nanog either through Oct4-Sox2, or through Oct4-Sox2-Nanog. To reach the stem cell state, Nanog has to be induced, as indicated by the red arrow. However, similar curves in the panels in the right columns in Figure 5, for the alternative path (ii), *i.e*, when *S_N_* crosses a certain threshold, Nanog comes on, and Gata-6 turns off. Activation of Nanog leads to reinforcement of the Oct4/Sox2/Nanog sub-network, due to their self-interactions, as well as suppression of Gata-6/Gcnf. Hence, the network reinforces itself, and the system is reprogrammed into the stem cell state. Notice the switch-like state, which is due to the positive feedbacks between the pluripotent genes *i.e.* as Nanog is activated, the trio Oct4-Sox2-Nanog reinforce each other by feeding back on each other positively, which gives the system co-operativity and hence bistable behavior [11]. An important point is that, although Nanog levels jump as *S_N_* increases, on removal of the Nanog activating signal, *S_N_* the system returns to the *endoderm* state, (there are two turning points in the plot). In the example shown, *A* = 25, and hence, according to Figure S1, the default state is the endoderm state. However, referring to Figure S1, *A* = 10, is in the bistable regime, and now if the initial condition is the endoderm state, inducing Nanog leads to a stem cell state. This can be seen in Figure S2, where removal of *S_N_* (after induction to the stem cell state) does not lead to the *endoderm* state (there is only one turning point in the curve).

## Discussion

We have developed a dynamical model for lineage determination: stem cell, *trophectoderm* and *endoderm*, for a network whose components are extracted from ChiP-on-chip and microarray data as well as literature studies [9]. This network exhibits some well known architectural motifs, such as autoregulation and mutual antagonism, which give rise to interesting dynamics. However, a visual inspection of the network is not sufficient to reveal its function, and hence this study provides an example of where mathematical modeling can help to quantify intuition.

In earlier work [11] we explored the dynamics of the core network of Oct4, Sox2 and Nanog, which is considered to be responsible for pluripotency/stemcellness [6], [7]. We demonstrated that positive feedbacks within this self-organized system gives rise to a bistable switch-like behavior, where the on state is the stem cell state and the off state is the differentiated state. Here we extend the model by including more components such as Cdx2, Gata-6 and Gcnf. The previously described differentiated state is now further refined into the *trophectoderm* and *endoderm* lineages.

One important ingredient in building our model is to address results obtained through microarray experiments [14]. Here, the authors uncovered the peculiar feature that a large fraction of genes responsible for stemcellness as well as differentiation are regulated in a biphasic manner with respect to Oct4 concentration levels. In trying to model this aspect of the network dynamics, we made two assumptions : (i) Oct4 positively activates Gata-6. This is required since over-expression of Oct4 must be able to turn Gata-6 on. (ii) Nanog is repressed by a heterodimer consisting of the Oct4–Gata-6 complex. This is required since, when Oct4 is over-expressed and Gata-6 is required to be turned on, Nanog is also induced. Therefore, a possible way to shut Nanog off, as Oct4 continues to increase, is to have it suppressed by the Oct4–Gata-6 complex.

The model successfully describes the various lineages in terms of the key transcription factor combinations, which naturally divide into three different regimes. (i) *Trophectoderm:* low levels of Oct4,Sox2 and Nanog, high levels of Cdx2, Gata-6. (ii) Stem cell: high levels of Oct4, Sox2 and Nanog. (iii) *Endoderm:* High levels of Oct4 and high levels of Gata-6. The “bell/inverted bell shaped curves” exhibited by these master regulators ensure that all their downstream target genes also show similar dynamics. Hence, this constrains binding mechanisms by which downstream target genes that are mutually regulated by Oct4/Sox2/Nanog, Gata-6, Gcnf and Cdx2, such that they too exhibit the biphasic behavior.

One outcome of the network dynamics is that the stem cell state must be the default state (see Figure S1), since this state cannot be reached from any of the other two states unless an external perturbation is applied (see the discussion regarding Figure S2 ). However, if the system starts in the stem cell state, then it is possible to transition into either of the two states, *trophectoderm*/*endoderm*, by decreasing/increasing Oct4 levels through external factors. To understand how a differentiated cell can be reprogrammed, we considered a specific example: reprogramming the cell to transition from the *endoderm* lineage to the stem cell state. We found that activating Nanog is a more robust way to reprogram the state, than by suppressing the genes (an example being Gata-6) responsible for differentiation. This is consistent with the model dynamics, since once the system falls from the “plateau”, the only way to re-establish the stem cell state, is to restart the self-organized pluripotency network. Once active, this would automatically ensure suppression of the differentiated state.

Recent experiments show that Oct4 targets, in particular Jmjd2c is a Histone demethylase for methyl marks on H3 Lys9 [22], which in turn maintains accessibility of Nanog. Since Nanog itself is part of the pluripotency self-organized network, this then could provide further positive feedback on Oct4 [23]. Hence by directly inducing Nanog, one sidesteps the need to wait for Oct4 induction of Nanog, both through first opening up the chromatin and then by direct transcription. As future experiments further develop our notions of the key players and their interactions, we hope to enhance/modify the current model to better describe the stem cell state. This would also allow more perturbations to be explored, to reprogram the cell. In the future we plan to explore the effects of stochastic fluctuations and the role they play in providing cues for differentiation into different lineages.

## Materials and Methods

The mathematical model for the networks presented assume a thermodynamic model of gene regulation [24], [25], [26]. In this framework, the transcriptional rate of a gene is proportional to the occupancy, which can be computed through computing equilibrium values of TF's which are bound to the promoters of the genes being transcribed. In Supplementary S1, we describe details of how the transcriptional rates discussed in Eqs. 1,2 below, are derived in terms of a reaction scheme. The specific assumptions made in constructing the model are (i) Oct4, Sox2 and Nanog positively feedback on each other through the binding of the Oct4-Sox2 and Oct4-Sox2-Nanog heterodimers [11]. (ii) The Oct4-Cdx2 heterodimer are repressors on both Oct4 and Cdx2, where the latter activates itself through the binding of Cdx2 to its own promoter [12]. (iii) Gcnf is activated by Cdx2 *or* Gata-6 [9]. It further suppresses Oct4, by binding to it as a repressor [17]. (iv) We assume that Nanog binds to Gata-6 as a repressor and Oct4 activates Gata-6 (both interactions are present in ChIP-chip data) [6], [19]. (v) the heterodimer Oct4–Gata-6, represses Nanog. For the effective model describing the architecture in Fig. 2 the equations for Nanog and Gata-6 are given by,
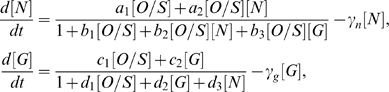
(1)Here, the concentrations of Nanog and Gata-6 are denoted [N] and [G] respectively and [O/S] denotes the concentration of the Oct4 and/or Oct4-Sox2 complex. Parameter values are found in Table 1. The corresponding model for the full network in Figure 1 is given by
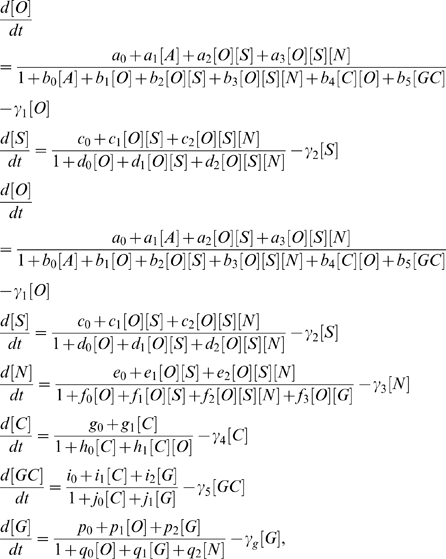
(2)where the parameter values are given in Table 2. The concentrations of Oct4, Sox2, Nanog, Cdx2, Gcnf, Gata-6 and the external signal ***A*** impinging upon Oct4 are denoted [O], [S], [N], [C], [GC], [G] and [A] respectively. We assume that the concentrations are dimensionless and the kinetic constants are in inverse time.

**Table 1:** Parameter values used in Figure 3.

*a*_1_	*a*_2_	*b*_1_	*b*_2_	*b*_3_	*γ_n_*	*c*_1_	*c*_2_	*d*_1_	*d*_2_	*d*_3_	*γ_g_*
0.02	0.0125	0.02	0.0125	0.03	0.01	0.05	0.0125	0.05	0.0125	0.05	0.01

**Table 2:** Parameter values used in Figure 4, Figure S1 and Figure S2.

*a*_0_	*a*_1_	*a*_2_	*a*_3_	*b*_0_	*b*_1_	*b*_2_	*b*_3_	*b*_4_	*b*_5_	*γ*_1_	*c*_0_
0.001	1.0	0.005	0.025	1.0	0.001	0.005	0.025	10	10	0.1	0.001
*c*_1_	*c*_2_	*d*_0_	*d*_1_	*d*_2_	*γ*_2_	*e*_0_	*e*_1_	*e*_2_	*f*_0_	*f*_1_	*f*_2_
0.005	0.025	0.001	0.005	0.025	0.1	0.001	0.1	0.1	0.001	0.1	0.1
*f*_3_	*γ*_3_	*g*_0_	*g*_1_	*h*_0_	*h*_1_	*γ*_4_	*i*_0_	*i*_1_	*i*_2_	*j*_0_	*j*_1_
10	0.1	0.001	2	2	5	0.1	0.001	0.1	0.1	0.1	0.1
*γ*_5_	*p*_0_	*p*_1_	*p*_2_	*q*_0_	*q*_1_	*q*_2_	*γ*_g_				
0.1	0.1	1.0	2.5 10^−4^	1.0	2.5 10^−4^	15	0.1				

The reprogramming studies of Figure 5, use the same parameters as in Table 2 with the following additional changes to Eq. 2. For the curves in the left column of Figure 5, where an external signal *S_G_* suppresses Gata-6, the denominator in the expression for *d*[*G*]/*dt* is appended with the term *q*_3_ *S_G_* with *q*_3_ = 10. Correspondingly for the right column of Figure 5, where Nanog is induced by an external signal *S_N_*, the expression for *d*[*N*]/*dt* is modified, by appending to its rate law, *i.e*, its numerator and denominator the terms *e*_3_ *S_N_* and *f*_4_ *S_N_*, respectively, with *e*_3_ = 1.0, *f*_4_ = 1.0. For both cases the external signal **A** = 25. For Figure S2, we use the same parameter values as in Table 2, but for A = 10.

## Supporting Information

Supplementary S1 In the supplementary information we describe (1)The origin of the rate equations used in the main text. (2) Two supplementary figures which support the main text(3) A simple model which implements squelching, with three figures.
(0.27 MB DOC)

Table S1 Parameter values used for Figure S4 and Figure S5.
(0.05 MB DOC)

Figure S1 Steady state concentrations of Oct4, Sox2, Nanog, Cdx2, Gata-6 and Gcnf as functions of the external signal A.
(0.04 MB EPS)

Figure S2 Steady state concentrations of Oct4, Nanog and Gata-6 as functions of external signals suppressing GATA-6 and activating Nanog respectvely.
(0.02 MB EPS)

Figure S3 A network schematic which implements a squelching mechanism by which Oct4 activates a target gene in a biphasic manner.
(0.07 MB EPS)

Figure S4 Steady state concentrations of X, target gene and complex C (Oct4-X) as functions of a term proportional to the Oct4 concentration.
(0.03 MB EPS)

Figure S5 Steady state concentrations of X, target gene and complex C (Oct4-X) as functions of a term proportional to the Oct4 concentration, without Oct4 regulation of co-factor X.
(0.09 MB EPS)

## References

[1] Takahashi K, Yamanaka S (2006). Induction of pluripotent stem cells from mouse embryonic and adult fibroblast cultures by defined factors.. Cell.

[2] Takahashi K, Tanabe K, Ohnuki M, Narita M, Ichisaka T (2007). Induction of pluripotent stem cells from adult human fibroblasts by defined factors.. Cell.

[3] Park IH, Zhao R, West JA, Yabuuchi A, Huo H (2008). Reprogramming of human somatic cells to pluripotency with defined factors.. Nature.

[4] Yu J, Vodyanik MA, Smuga-Otto K, Antosiewicz-Bourget J, Frane JL (2007). Induced pluripotent stem cell lines derived from human somatic cells.. Science.

[5] Meissner A, Wernig M, Jaenisch R (2007). Direct reprogramming of genetically unmodified fibroblasts into pluripotent stem cells.. Nat Biotechnol.

[6] Boyer LA, Lee TI, Cole MF, Johnstone SE, Levine SS (2005). Core transcriptional regulatory circuitry in human embryonic stem cells.. Cell.

[7] Loh Y-H, Wu Q, Chew J-L, Vega VB, Zhang W (2006). The Oct4 and Nanog transcription network regulates pluripotency in mouse embryonic stem cells.. Nat Gen.

[8] Ivanova N, Dobrin R, Lu R, Kotenko I, Levorse J (2006). Dissecting self-renewal in stem cells with RNA interference.. Nature.

[9] Niwa H (2007). How is pluripotency determined and maintained?. Development.

[10] Johnson BV, Rathjen J, Rathjen PD (2006). Transcriptional control of pluripotency: decisions in early development.. Curr Opin Genet Dev.

[11] Chickarmane V, Troein C, Nuber UA, Sauro HM, Peterson C (2006). Transcriptional dynamics of the embryonic stem cell switch.. PLoS Comput Biol.

[12] Niwa H, Toyooka Y, Shimosato D, Strumpf D, Takahashi K (2005). Interaction between Oct3/4 and Cdx2 determines *trophectoderm* differentiation.. Cell.

[13] Ralston A, Rossant J (2005). Genetic regulation of stem cell origins in the mouse embryo.. Clin Genet.

[14] Matoba R, Niwa H, Masui S, Ohtsuka S, Carter MG (2006). Dissecting Oct3/4-Regulated gene networks in embryonic stem cells by expression profiling.. PLoS ONE.

[15] Nichols J, Zevnik B, Anastassiadis K, Niwa H, Klewe-Nebenius D (1998). Formation of pluripotent stem cells in the mammalian embryo depends on the POU transcription factor Oct4.. Cell.

[16] Niwa H, Miyazaki J, Smith AG (2000). Quantitative expression of Oct-3/4 defines differentiation, dedifferentiation or self-renewal of ES cells.. Nat Gen.

[17] Gu P, LeMenuet D, Chung AC, Mancini M, Wheeler DA (2005). Orphan nuclear receptor GCNF is required for the repression of pluripotency genes during retinoic acid-induced embryonic stem cell differentiation.. Mol Cell Biol.

[18] Mullen EM, Gu P, Cooney AJ (2007). Nuclear receptors in regulation of mouse ES cell pluripotency and differentiation.. PPAR Res.

[19] Boyer LA, Mathur D, Jaenisch R (2006). Molecular control of pluripotency.. Curr Opin Genet Dev.

[20] Schöler HR, Ciesiolka T, Gruss P (1991). A nexus between Oct-4 and E1A: implications for gene regulation in embryonic stem cells.. Cell.

[21] Hay DC, Sutherland L, Clark J, Burdon T (2004). Oct-4 knockdown induces similar patterns of endoderm and trophoblast differentiation markers in human and mouse embryonic stem cells.. Stem Cells.

[22] Loh YH, Zhang W, Chen X, George J, Ng HH (2007). Jmjd1a and Jmjd2c histone H3 Lys 9 demethylases regulate self-renewal in embryonic stem cells.. Genes Dev.

[23] Niwa H (2007). Open conformation chromatin and pluripotency.. Genes Dev.

[24] Buchler NE, Gerland U, Hwa T (2003). On schemes of combinatorial transcription logic.. Proc Natl Acad Sci USA.

[25] Bintu L, Buchler NE, Garcia HG, Gerland U, Hwa T (2005). Transcriptional regulation by the numbers: models.. Curr Opin Genet Dev.

[26] Hasty J, Isaacs F, Dolnik M, McMillen D, Collins JJ (2001). Designer gene networks: Towards fundamental cellular control.. Chaos.

