# Supplementary material for: A Computational Model for Understanding Stem Cell, Trophectoderm and Endoderm Lineage Determination
Source: PLoS One. 2008 Oct 22;3(10):e3478. doi: 10.1371/journal.pone.0003478 (PMC2566811; doi:10.1371/journal.pone.0003478)
Supplement: Supplementary S1 — In the supplementary information we describe (1)The origin of the rate equations used in the main text. (2) Two supplementary figures which support the main text(3) A simple model which implements squelching, with three figures. (0.27 MB DOC) [file pone.0003478.s001.doc]

**Supporting Information**

**A computational model for understanding**

**stem cell, trophectoderm and endoderm lineage determination**

**Vijay Chickarmane and Carsten Peterson**

**1. Derivation of Eqs. 1, 2 in the** *Main Text*

Here we discuss the structure of Eqs. 1, 2 in the *Main Text*. It is possible to separate the evolution of the transcription factor (TF) concentrations into two time scales [1]: a fast time scale, if we assume rapid kinetics of protein TF-DNA binding, and a slow time scale, that of the processes of transcription and degradation. Moreover we will assume that the binding-unbinding reactions are so fast that we can use their equilibrium concentrations to compute the gene occupancy. Below we describe the reaction schemes which give rise to Eqs. 1, 2.

**Eq. 1 -- A network description of the "bell shaped" curve**

From the *Main Text*, we denote the concentrations of Oct-Sox, Nanog and Gata-6 by $[O/S]$, $[N]$ and $[G]$ respectively. Representing $g_{N}^{i}$ as the fractional occupancy of the Nanog promoter by species $i$, one gets,

(S1)

Since $\sum g_{N}^{i}=g_{T}$ (total concentration of DNA promoter sites is conserved, furthermore, we assume $g_{T}=1$) , and assuming the equilibrium values,  *i.e.* $g_{N}^{1}=k_{1}g_{N}^{0}[O/S]$, we obtain

$g_{N}^{0}=\frac{g_{T}}{1+k_{1}[O/S]+k_{1}k_{2}[O/S][N]+k_{1}k_{3}[O/S][G]}$ (S2)

Nanog is transcribed by RNA polymerase (RNAP) when it is bound either by $[O/S]$ or by $[O/S][N]$. Simplifying the processes of transcription and translation into one combined process, we get,

$g_{N}^{1}\to g_{N}^{1}+[N]; \alpha_{1}$ (S3)

$$g_{N}^{2}\to g_{N}^{1}+[N]; \alpha_{2}$$

from which we get for the production rate of $[N]$, $T$ which is proportional to $\alpha_{1}g_{N}^{1}+\alpha_{2}g_{N}^{2}$ is,

$T=\frac{k_{1}\alpha_{1}[O/S]+k_{1}k_{2}\alpha_{2}[O/S][N]}{1+k_{1}[O/S]+k_{1}k_{2}[O/S][N]+k_{1}k_{3}[O/S][G]}$ (S4)

The functional form of $T$ is similar to the first term used in Eq. 1 (Main text), for $d[N]/dt$, which is,

$\frac{a_{1}[O/S]+a_{2}[O/S][N]}{1+b_{1}[O/S]+b_{2}[O/S][N]+b_{3}[O/S][G]}$ (S5)

The reaction scheme to obtain the transcription rate for $d[G]/dt$ is,

(S6)

where we assume that Gata-6 can be transcribed when it is in the states $g_{G}^{1}$ and $g_{G}^{2}$. With similar assumptions used in deriving Eq. S3, we obtain for Gata-6

$T=\frac{k_{3}\alpha_{1}[O/S]+k_{5}\alpha_{2}[G]}{1+k_{3}[O/S]+k_{5}[G]+k_{6}[N]}.$ (S7)

Once again it is functionally similar to first term in $d[G]/dt$, Eq. 1 (Main text).

**Eq. 2-- The stem cell network.**

In what follows we simply state the reaction scheme used to generate the transcription rates for Eq. 2 (Main text), for each of the equations.

•  **Oct4**

(S8)

Oct4 is transcribed when bound by $[A]$, or $[O/S]$ or $[O/S][N]$. We also assume that there is a low basal rate at which the Oct4 promoter can be bound to RNAP in the absence of any TF's, hence $T=\alpha_{0}g_{O}^{0}+\alpha_{1}g_{O}^{1}+\alpha_{3}g_{O}^{3}+\alpha_{4}g_{O}^{4}$

•  **Sox2**

(S9)

Sox2 is transcribed when bound by, $[O/S]$ or $[O/S][N]$, with a low basal rate, hence $T=\alpha_{0}g_{S}^{0}+\alpha_{2}g_{S}^{2}+\alpha_{3}g_{S}^{3}$.

•  **Nanog**

(S10)

Nanog is transcribed when bound by, $[O/S]$ or $[O/S][N]$ with a low basal rate, hence $T=\alpha_{0}g_{N}^{0}+\alpha_{2}g_{N}^{2}+\alpha_{3}g_{N}^{3}$.

•  **Cdx2**

(S11)

Cdx2 is transcribed when bound by, $[C]$, and a low basal rate such that $T=\alpha_{0}g_{C}^{0}+\alpha_{1}g_{C}^{1}$.

•  **Gcnf**

(S12)

Gcnf is transcribed when bound by, $[G]$ or $[C]$, with a low basal rate such that, $T=\alpha_{0}g_{GC}^{0}+\alpha_{0}g_{GC}^{1}+\alpha_{2}g_{GC}^{2}$.

•  **Gata-6**

(S13)

Gata-6 is transcribed when bound by, $[O]$ or $[G]$ with a low basal rate, hence $T=\alpha g_{G}^{0}+\alpha g_{O}^{1}+\alpha_{2}g_{G}^{2}$.

**2. Steady state values of network component concentrations**

**as functions of *A***

In Figure S1 the steady state concentrations of Oct4, Sox2, Nanog, Cdx2, Gata-6 and Gcnf are shown as functions of the external signal $A$ (see  *Main Text*). There are three dynamical regions, between the two saddle-nose bifurcation points (SN). The borders between the regions are marked by red arrows. The first region occurs for $A<6$, the second between $6<A<20$, and the third for $A>20$. The three regions correspond to the  *trophectoderm*, embryonic and  *endoderm* lineages respectively. Note that the middle region is bistable, and hence the embryonic state is one of two options for this range of external signal A.

**3. Steady state values of network component concentrations**

**as functions of** $N_{G}$

In Figure S2 the steady state concentrations of Oct4, Nanog, and Gata-6 are shown as functions of the external signal $S_{N}$ (see Figure 5  *Main Text*), which activates Nanog. We have assumed that the external signal $A=10$, and that the starting state is the endoderm state. As Nanog builds up due to activation by $S_{N}$, it reaches a threshold at which the positive feedbacks between Oct4, Sox2 and Nanog reinforce each other, and the system "jumps" to the embryonic state. Once in the embryonic state, the system is fully committed, since on removal of the signal $S_{N}$, the system does not fall back to its original, endoderm state. This is evident from the fact that here is only one turning point (SN) in the plot . From Figure S1, it is now clear that the system has been re-programmed by toggling the bistable regime ($A=10$ which puts the system exactly in this regime) through a perturbation in Nanog.

**4. Modeling a squelching mechanism**

We discuss a simple model of gene regulation implementing the squelching mechanism [2], which aims at describing the biphasic response of Oct4. Let us assume that a target gene is regulated by Oct4 and a co-factor X. Also assume that Oct4 or X by itself can bind to the target gene (TG), but cannot by themselves initiate transcription. However, the Oct4-X heterodimer can positively activate the target gene. Moreover, we also assume that X is negatively regulated by Oct4. The network which emerges from these assumptions is drawn in Figure S3, where the red line illustrates the hypothesis. The corresponding equations are given by

$\frac{d[O]}{dt}=a_{1}-\gamma_{1}[O]-k_{1}[O][X]+k_{2}[C]$ (S14)

$$\frac{d[X]}{dt}=\frac{\alpha_{1}}{1+\alpha_{2}[O]^{n}}-\gamma_{2}[X]-k_{1}[O][X]+k_{2}[C]$$

$$\frac{d[TG]}{dt}=\frac{\delta_{1}[C]}{1+\delta_{2}[C]+\delta_{3}[O]+\delta_{4}[X]}-\gamma_{3}[TG]$$

$$\frac{d[C]}{dt}=k_{1}[O][X]-k_{2}[C]$$

Where [O], [X], [TG] & [C] refer to the concentrations of Oct4, X, Target gene and the complex Oct4-X, and the parameter values are given in Table S1 below.

In Figure S4 we show the biphasic behavior of the target gene as well as for X. For low values of Oct4, there is a low amount of the complex which can therefore only weakly activate the target gene. For high levels of Oct4, assuming that X is repressed, once again the amount of activating complex is low. However, for intermediate levels of Oct4, the complex reaches a maximum, and hence can activate the target gene maximally. In Figure S5, where we assume that $\alpha_{2}=0$ (Eq. S14), the effect of removing the Oct4 suppression of X is seen. Since X is not controlled by Oct4, it is always available to form a complex with Oct4. Hence the complex levels keep increasing with the Oct4 concentration. whereas the target gene expression saturates, it does not display the "bell shaped" behavior. We infer from this that a squelching mechanism by itself would be unable to give the biphasic response. However, if in addition to this mechanism we assume that the co-factor which partakes in the squelching mechanism is regulated by Oct4, then we obtain the biphasic behavior.

**Simulations**

Simulations were implemented using MATLAB (The Mathworks), JDesigner (SBW) [3] and Oscill8 (bifurcation software)[4].

**References**

[1] Hasty J, Isaacs F, Dolnik M, McMillen D, Collins JJ. (2001) Designer gene networks: Towards fundamental cellular control.  *Chaos*. 1:207-220.

[2] Schöler HR, Ciesiolka T, Gruss P. (1991) A nexus between Oct-4 and E1A: implications for gene regulation in embryonic stem cells.  *Cell.* 66:291-304.

[3] Sauro, H. M., Hucka, M., Finney, A., Wellock, C., Bolouri, H., Doyle, J., & Kitano, H.).(2003) Next generation simulation tools: the systems biology workbench and biospice integration.  *OMICS.* 7:355-372.

[4] http://sourceforge.net/projects/oscill8

**FIGURES**

Figure S1: Steady state concentrations of Oct4, Sox2, Nanog, Cdx2, Gata-6 and Gcnf as functions of the external signal $A$.

Figure S2: Steady state concentrations of Oct4, Nanog and Gata-6 as functions of the external signal $S_{N}$. The system is re-programmed to the embryonic state through Nanog perturbation.

Figure S3: The network for a squelching mechanism of Oct4. A complex C of Oct4 and X is formed, which positively regulates the target gene. Individually, Oct4 and X each negatively regulate the target gene, and the dashed red line hypothesizes negative a regulation of X by Oct4.

Figure S4:The steady state concentrations of X, the target gene and the complex C (Oct4-X) as a functions of a term $a_{1}$ (Eq. S14) proportional to the Oct4 concentration. Assuming that Oct4 suppresses X, a "bell shaped" curve for the expression level of the target gene/complex is obtained.

Figure S5: The steady state concentrations of X, the target gene and the complex C (Oct4-X) as functions of a term $a_{1}$ (Eq. S14) proportional to the Oct4 concentration. Since X is not regulated in any way by Oct4, the complex continues to grow. Meanwhile the target gene expression saturates, but does not exhibit the "bell shaped" curve.
