# Supplementary material for: A Computational Model for Understanding Stem Cell, Trophectoderm and Endoderm Lineage Determination
Source: PLoS One. 2008 Oct 22;3(10):e3478. doi: 10.1371/journal.pone.0003478 (PMC2566811; doi:10.1371/journal.pone.0003478)
Supplement: Table S1 — Parameter values used for Figure S4 and Figure S5. (0.05 MB DOC) [file pone.0003478.s002.doc]

Table S1

| $a_{1}$ | $\alpha_{1}$ | $\alpha_{2}$ | $n$ | $k_{2}$ | $k_{1}$ | $\delta_{1}$ | $\delta_{2}$ | $\delta_{3}$ | $\delta_{4}$ |
| --- | --- | --- | --- | --- | --- | --- | --- | --- | --- |
| $0.04$ | 0.5 | 1.0 | $2$ | 0.5 | 0.1 | 1 | 1.0 | 0.1 | 0.1 |

Table S1: Parameter values used for Figure S4 and Figure S5, with $\gamma_{i}=0.01$, for $i=1:3$. For Figure S5, $\alpha_{2}=0$.
